# Update on Neuropathies in Inborn Errors of Metabolism

**DOI:** 10.3390/brainsci11060763

**Published:** 2021-06-08

**Authors:** Renata Pająk, Ewelina Mendela, Natalia Będkowska, Justyna Paprocka

**Affiliations:** 1 Students’ Scientific Society, Department of Pediatric Neurology, Faculty of Medical Sciences in Katowice, Medical University of Silesia, 40-752 Katowice, Poland; renia.pajak@gmail.com (R.P.); ewelinamendela97@gmail.com (E.M.); nataliabedkowska7@gmail.com (N.B.); 2 Department of Pediatric Neurology, Faculty of Medical Sciences in Katowice, Medical University of Silesia, 40-752 Katowice, Poland

**Keywords:** inborn errors of metabolism, peripheral neuropathy, Fabry Disease, porphyria, small fiber neuropathy

## Abstract

Neuropathies are relatively common in inborn errors of metabolism (IEMs); however, due to the early onset and severe, progressive course of many IEMs, they have not been very well researched yet. This article aims to review and compare neuropathies in inborn errors of metabolism, mostly with childhood and juvenile onset. Some of these diseases are treatable if diagnosed early and in many cases, the therapy can not only slow down disease progression, but can also reverse the changes already made by the condition.

## 1. Introduction

The term neuropathy refers to all conditions in which damage to a peripheral nerve causes different symptoms depending on its function. The most common symptom is usually ice-burn-like neuropathic pain. Other pain-associated manifestations may involve paraesthesia, allodynia and hyperalgesia. Additionally, damage to a motor nerve may result in involuntary muscle contractions, twitching or muscle weakness and reflex abnormalities, while sensory nerve damage might lead to loss of sensation, numbness, paraesthesia and gait-ataxia. An impairment of autonomic nerve function can cause cardiac arrhythmias, constipation, vomiting, urinary incontinence, tachycardia, excessive sweating or orthostatic hypotension. Since affected nerves may carry fibers with different functions, neuropathies are often combined—sensorimotor being the most common variant. The symptoms often have a characteristic stocking-glove distribution, affecting mostly distal parts of both upper and lower extremities. Neuropathies can be divided, depending on their course, into acute and chronic. They can also be categorized into mononeuropathies, multifocal neuropathies and polyneuropathies of either demyelinating or axonal kind. This classification, however, refers only to large fibers, which is why the small fiber neuropathies present, among others, in Fabry disease, one of the most common IEMs, will be covered separately [1,2,3]. A classification of neuropathy types in different IEMs can be found in Table 1.

## 2. Acute Neuropathies

Acute neuropathy is a rapid, progressive disease, with symptoms usually lasting less than 4 weeks. It is most commonly caused by the autoimmune-associated Guillain–Barre syndrome (GBS), but can also be triggered by different kinds of toxins especially by heavy metals, as well as by infections, the most common ones being Lyme disease and diphtheria, other autoimmune diseases, including vasculitis, or by critical illnesses. They can also be caused by severe vitamin B1 deficiency or occur due to a paraneoplastic syndrome [3,4]. Only few of them might however be caused by genetic factors, both hereditary neuropathies and inborn errors of metabolism. The most common IEM presenting with such symptoms is acute intermittent porphyria (AIP), a defect of heme synthesis enzymes leading to excessive accumulation and excretion of porphyrins and their precursors, including neurotoxic δ-aminolevulinic acid (ALA) and porphobilinogen (PBG). Other kinds of porphyria presenting with neurological symptoms include HCP (hereditary coproporphyria), variegate porphyria and ALA (aminolevulinic acid) dehydratase deficiency porphyria.

Severe porphyric neuropathy can be isolated or occur simultaneously with other AIP symptoms. It manifests itself as a combination of autonomic and peripheral neuropathy, the latter one usually beginning after the onset of the characteristic abdominal pain, psychiatric abnormalities and other CNS symptoms. It is generally considered to be a primarily motor neuropathy, often with asymmetric presentation. Disease progression can lead to quadriparesis and respiratory problems [5,6,7].

Tyrosinaemia type 1 can also present with acute symptoms, especially in untreated patients. The symptoms include episodes of pain and/or paralysis, in some cases with hypertonic posturing, resembling opisthotonus [8].

Another IEM which can have both acute and chronic presentation is Classic Refsum Disease. Its most distinct features are retinitis pigmentosa and anosmia, often accompanied by ataxia and usually chronic polyneuropathy. In rare cases it can, however, present with acute deterioration, usually triggered by weight loss, stress, trauma or infections. It is characterized by a mixed sensory-motor polyneuropathy which, if untreated, can lead to muscular atrophy and weakness of lower extremities, as well as of the trunk [9,10].

Among its many phenotypes, pyruvate dehydrogenase (PDH) deficiency can also cause sudden weakness, especially during an infection [11]. Sporadically, IEMs causing chronic neuropathies can also cause exacerbations and present with symptoms of acute neuropathy. There have been reports of Leigh syndrome presenting with sudden weakness and GBS-like symptoms, leading to quadriplegia and the patient requiring mechanical ventilation [12,13].

## 3. Mononeuropathy and Multifocal Neuropathy

Unlike in adults, mononeuropathies in childhood are most commonly caused by traumatic nerve damage, and much less often by compressive lesions or by entrapment. The location also differs from the adult neuropathies, with median, peroneal, ulnar and radial and sciatic nerves equally often affected. Carpal tunnel syndrome is relatively rare in children; its presence is therefore suggestive of mucolipidosis or mucopolysaccharidosis, and it also can appear in as many as 1 in 4 young patients with Fabry Disease. Among the genetic causes, hereditary polyneuropathy with liability to pressure palsy should be considered in adolescents and young adults, as well as other inborn errors of metabolism [14,15].

Mucopolysaccharidoses are a group of rare lysosomal storage diseases caused by the accumulation of glycosaminoglycans (GAGs) in tissues. They present with a variety of symptoms, such as skeletal and joint dysplasia, corneal clouding and other ophthalmological findings, coarse facial features, hernias and heart valve disease. They can also have variable neurological manifestations, that can range from carpal tunnel syndrome to psychomotor retardation, cognitive dysfunction, compression neuropathy, waddling gait and hearing loss. Depending on the enzyme affected, seven different subtypes can be distinguished. Carpal tunnel syndrome is especially prevalent in patients with type I (Hurler Syndrome), II (Hunter Syndrome), IV (Morquio syndrome) and VI (Maroteaux–Lamy syndrome), which justifies frequent screening (physical examination and obtaining history for carpal tunnel syndrome every 6 months from the diagnosis, in some cases annual nerve conduction studies) in the affected patients [16,17].

One of the other rare IEMs presenting with such symptoms is Tangier disease, where focal and multifocal sensory and motor neuropathies account for, respectively, 26.2% and 19.1% of all neuropathies [18].

## 4. Chronic Axonal Polyneuropathies

Chronic axonal polyneuropathy, the most common type of all, is usually induced by toxic and metabolic conditions, especially by diabetes mellitus, chronic renal insufficiency and complications of chemotherapy. Other important causes include nutritional deficiencies, especially of thiamin, vitamin B12, gluten sensitivity and other cancer-related and genetic causes, including hereditary neuropathies and IEMs [19]. Axonal neuropathy typically manifests as a symmetric sensory loss in the upper and/or lower extremities with a characteristic stocking-glove distribution. Proximal parts of limbs are usually affected later in the course of the disease [1]. Among others, the IEMs most commonly presenting with this neuropathy type are mitochondrial disorders, above all respiratory chain diseases—NARP, Leigh syndrome and LHON [4].

These conditions are characterized by maternal inheritance and a phenomenon called heteroplasmy which indicates the presence of different extranuclear DNA types in a cell. This leads to different phenotypes depending on the quantity of mutated DNA, for example 70–90% of mitochondrial genes are affected with a specific mutation results in NARP-Syndrome (Neuropathy, Ataxia, Retinitis Pigmentosa), while more than 90% affected manifests as Leigh Syndrome. NARP is caused by mitochondrial DNA mutations, altering the energy translation in complex V and thus destabilizing 6. subunit of ATPase. It presents with sensory manifestations including retinitis pigmentosa and a sensorineural hearing loss, as well as with neurological symptoms including neuropathy, epileptic seizures or weakness and ataxia [20]. Both EMG and nerve conduction studies show a peripheral neuropathy of sensory axonal or sensorimotor axonal type.

Leigh syndrome is a frequent manifestation of many mitochondrial diseases with the onset usually in early childhood, often following a viral infection. Neurological symptoms include muscle hypotonia or spasticity, regression of psychomotor development, movement disorders, seizures, ophthalmologic manifestations, cerebellar ataxia and peripheral sensory neuropathy. Other manifestations including cardiomyopathy (one of the most frequent causes of death), diabetes, short stature and many other features that account for the Leigh-like syndrome [12,21].

LHON (Leber’s Hereditary Optic Neuropathy) is caused by a mitochondrial DNA mutation resulting in structural changes of the respiratory chain complex I. It usually affects relatively older patients, mostly young adults. The most common symptom is a severe optic neuropathy, usually characterized by subacute bilateral vision loss. It may be triggered by vitamin deficiencies, or alcohol, tobacco and/or drug abuse [22]. Pathological studies of the brachial plexus/a brachial plexus biopsy in LHON patients show a neurodegenerative pattern. Other manifestations may include subclinical peripheral or progressive auditory neuropathy. This and other non-ophthalmologic manifestations can be referred to as “Leber’s plus” clinical phenotype [23].

Another condition presenting with retinitis pigmentosa and chronic axonal polyneuropathy is a-methylacyl-CoA racemase deficiency [24].

The most prevalent mitochondrial mutation leading to neurological manifestations is POLG gene mutation. It has six major manifestation forms and two of them include neuropathies—ataxia neuropathy spectrum (ANS) and autosomal dominant progressive external ophthalmoplegia (Ad PEO). ANS is a new term which includes sensory ataxic neuropathy with dysarthria and ophthalmoplegia (SANDO) and mitochondrial recessive ataxia syndrome (MIRAS). POLG mutations present with predominantly sensory neuropathy, usually axonal or mixed [25].

Serine deficiency can manifest at any age, the symptoms may, however, vary, with absence seizures and psychomotor retardation present more often in the early onset patients and progressive sensorimotor neuropathy affecting mostly patients with a late onset. It is caused by a mutation in one of the three enzymes responsible for its biosynthesis: 3-phosphoglycerate dehydrogenase (3-PGDH), phosphoserine aminotransferase or phosphoserine phosphatase. Méneret et al., 2012 reported of a 31-year old patient with severe progressive axonal sensorimotor neuropathy of adult onset, suspected of CMT type 2, who was eventually diagnosed with 3-PDGH deficiency. The patient also had congenital cataracts, mild walking difficulties and mental retardation in childhood. Other findings included cerebellar ataxia and nystagmus [26].

Early manifestation may suggest a MTP (mitochondrial trifunctional protein) or LCHAD (long-chain 3-hydroxyacyl-CoA dehydrogenase) deficiency, conditions presenting with a chronic sensorimotor polyneuropathy in the initial stadium, which can later be accompanied by episodic rhabdomyolysis. Although both diseases can show signs of polyneuropathy and retinopathy, it should be noted that the first one appears more frequently in MTP patients, whereas the latter one is more characteristic for LCHAD deficiency [27].

A progressive early onset axonal neuropathy is also the main feature of Brown-Vialetto-van Laere syndrome (BVVL) and Fazio-Londe Syndrome (patients without hearing loss), also called riboflavin transporter deficiency. The mutations causing it are located in genes encoding the riboflavin transporters type 2 and 3. BVVL usually first affects cranial nerves and can present as sensorineural hearing loss, optic atrophy or nerve palsy. Late symptoms may involve bulbar dysfunction with dysphagia and respiratory problems. The progressive axonal neuropathy, depending on its type can either affect upper extremities in type 2 (transporter deficiency) or cause generalized weakness in type 3. Type 2 is also characterized by sensory ataxia and ganglionopathy. This illness requires special attention, because in some cases it responds to riboflavin supplementation. Best results can be achieved with early beginning of the treatment [28,29].

A progressive sensory, motor and autonomic neuropathy can also be observed in patients with transthyretin-type familial amyloid polyneuropathy (ATTR-FAP). This condition can also present as restrictive cardiomyopathy and cerebral amyloid angiopathy. The onset is usually relatively late in life with early onset patients usually affected from late 20 s to early 40 s and late-onset ones above the age of 50. In early onset patients small fibers conducting pain and temperature sensation are usually first affected, leading to dysesthesias, paresthesias, allodynia and neuropathic pain in feet, then to a length-dependent progression. Manifestations in lower extremities tend to precede those in upper limbs, whereas in late-onset form the disease affects simultaneously arms and legs. Autonomic dysfunction is very severe in patients with early onset, the symptoms often include diarrhea, erectile dysfunction, orthostatic hypotension, etc. Other findings suggestive of ATTR-FAP include carpal tunnel syndrome, unexplained weight loss, family history of ATTR-FAP, gastrointestinal symptoms and cardiac involvement [30].

An isolated form of neuropathy can also appear in Wilson’s disease, in which it requires a particularly thorough examination, since it might also occur due to penicillamine intake or severe liver disease [31,32].

In CTX (cerebrotendinous xanthomatosis) an autosomal recessive leukodystrophy and vitamin E deficiency both cases of axonal and demyelinating neuropathies have been reported [4,33,34].

## 5. Chronic Demyelinating Neuropathies

Chronic demyelinating polyneuropathies are far less frequent than axonal ones and can be caused by autoimmune-induced myelin dysfunction. The diseases most commonly presenting with this neuropathy pattern are chronic inflammatory demyelinating polyradiculopathy (CIDP) and among the inherited neuropathies, Charcot–Marie–Tooth Disease, especially type 1, 4 and X1. Among the IEMs, it can be present in many lysosomal storage disease, including beta-mannosidosis, metachromatic leukodystrophy, Krabbe Disease and Niemann-Pick B disease [35].

β-mannosidosis often manifests with a wide phenotypic heterogeneity. Its symptoms may include developmental delay in various degrees of severity, behavioral abnormalities, frequent infections and hearing loss, as well as demyelinating peripheral neuropathy [36].

Rapidly progressing demyelinating neuropathy is the main feature of late-infantile MLD (metachromatic leukodystrophy). Its symptoms usually begin around the age of 2, often preceding other neurological symptoms. It is the most common MLD type, accounting for around 50 percent of all cases. The neuropathy is, however, not frequent in juvenile and adult MLD forms, which manifest with psychiatric symptoms and behavioral abnormalities, including alcohol and drug abuse [37].

Another lysosomal storage disease rarely presenting with demyelinating neuropathy is Niemann–Pick B disease, a condition which belongs to acid sphingomyelinase-deficient Niemann–Pick disease (ASMD) [38].

Chronic demyelinating polyneuropathy may be isolated or appear as one of a wide spectrum of symptoms. In case of a neuropathy presenting in 3–8 year old children, juvenile onset Krabbe disease should be considered [4]. It is far less common than the infantile type with the symptoms including psychomotor retardation, vision loss, seizures and peripheral neuropathy [39].

Classic Refsum disease is a peroxisomal disorder, caused by accumulation of phytanic acid in blood and tissue. Patients can exhibit mixed asymmetric polyneuropathy, which can cause weakness of lower extremities and progress to involve the trunk as well, the disease may also cause bilateral sensorineural hearing loss.

Another disease caused by phytanic acid accumulation is Infantile Refsum Disease (IRS). It is a part of the Zellweger spectrum disorders (ZSDs), showing multiple similarities to the two other diseases from this group, Zellweger syndrome and adrenoleukodystrophy, which can also manifest with peripheral neuropathy.

It can present already in 6 month old infants, leading to visual impairment, jaundice, failure to thrive and hypotonia as well as to neurological symptoms including mental retardation, sensorineural deafness, developmental delays, anosmia and peripheral neuropathy.

Like in Classic Refsum Disease, the treatment is based on the phytanic acid restriction and therapeutic plasma exchange. Due to its slow progression, the patients usually survive till adolescence and in some cases till young adulthood [9,40].

Demyelinating polyneuropathy can also be prevalent in homocysteine remethylation disorders—the adult form of cobalamin C disease (CblC) and MTHFR (methylenetetrahydrofolate reductase) deficiency causing gait-ataxia and psychiatric symptoms, often together with a cognitive decline (which are better responsive to treatment than early onset forms [41]), as well as in MNGIE (mitochondrial neurogastrointestinal encephalomyopathy) [42].

## 6. Small Fiber Neuropathy

Small fiber neuropathy is a result of damage to peripheral nerves, specifically Aδ or unmyelinated C fibers, responsible for pain and temperature perception, as well as for autonomic functions. Its symptoms include pain, usually situated in the hands and feet, paraesthesias, allodynia, and feelings of tingling or burning. Disorders involving autonomic fibers can result in abnormal sweating, tachycardia, vomiting, constipation or orthostatic hypotension. Symptoms may be mild in early stages; however, in some cases neuropathy is severe. Small fiber neuropathy can be caused by diabetes, impaired glucose tolerance, amyloidosis, hepatitis C, Sjorgen’s syndrome and many others [43,44].

An IEM most frequently presenting with these symptoms is Fabry disease, a lysosomal storage disorder with many other systemic manifestations, including renal failure, cardiac and cerebrovascular disease and skin lesions. In patients with the classic phenotype, the first symptoms include episodes of burning, tingling or shooting pain, angiokeratomas, and heat intolerance, and gastrointestinal symptoms can appear in early childhood, even in 6–8-year-olds [45,46]. Patients with the nonclassical phenotype are generally less severely affected, with symptoms usually limited to a single organ, and their progression is also slower than in the classic form. The diagnosis is usually made relatively late due to nonspecific early symptoms.

Patients may exhibit exercise intolerance and have acute neuropathic pain crises, presenting with extreme proximally radiating pain in the hands and feet. Symptoms may be triggered by various stimuli, especially by extreme temperatures, fatigue, stress or exercise [47].

Tangier disease is a familial high-density lipoprotein deficiency, of autosomal recessive inheritance. It results in the accumulation of cholesteryl esters in body tissues, which leads to different clinical symptoms, including enlarged, lipid-laden tonsils, hepatosplenomegaly and a peripheral neuropathy, of chronic or relapsing–remittent nature [48]. It can be further divided into four subtypes, the most common one being syringo-myelia-like neuropathy with symptoms first appearing in the face or arms, loss of pain and temperature sensation except in the feet, as well as a pronounced weakness in the arms. Multifocal sensory and motor neuropathy, accounting for approximately a quarter of all cases and focal neuropathy both demonstrate a relapsing–remitting pattern. Distal symmetric neuropathy, the rarest type, is either a stable or a progressive disease [18].

Gaucher Disease is the most common sphingolipidosis, caused by a mutation in the *GBA1* gene, leading to impaired beta-glucocerebrosidase activity and glucosylceramide accumulation. There are three types of the disease—type 1, which used to be described as a non-neuropathic form, although recent studies show progressive peripheral neuropathy of diverse intensity (in many cases subclinical) in some patients, presenting as mononeuropathy, sensory motor axonal polyneuropathy [49] or small fiber neuropathy [50]. Patients with type 3 Gaucher disease exhibit diverse neurological symptoms, from horizontal ophthalmoplegia to cerebellar ataxia, dementia or progressive myoclonus epilepsy. Type 2 is characterized by early and severe neurological symptoms—the classic triad involves bulbar signs, opisthotonus and oculomotor paralysis [51].

The early onset form of TTR familial amyloidosis (earlier described in chronic axonal neuropathies) usually begins as a small-fiber neuropathy affecting pain and temperature sensation in the feet, later progressing to involve whole legs and all kinds of perception. It is also characterized by extremely severe autonomic dysfunction, which if untreated can lead to death [30].

## 7. Lower Motor Neuron Disease

The term lower motor neuron disease refers to all diseases affecting spinal and bulbar motor neurons. Depending on its cause, it can be immune-mediated (multifocal motor neuropathy and CIDP), hereditary (SMAs, distal hereditary motor neuropathies) or sporadic. IEMs presenting with these kinds of symptoms are GM2-gangliosidosis, polyglucosan body disease and biotinidase deficiency [4].

GM2 gangliosidoses are lysosomal disease caused by GM2 ganglioside accumulation due to enzyme deficiency—depending on the enzyme affected—Tay–Sachs Disease (type I, hexosaminidase A affected), Sandhoff Disease (type II, hexosaminidases A and B affected) and deficiency of GM2-activator protein (very rare, sometimes referred to as AB variant) can be distinguished.

The symptoms and course of the disease depend on the time of its onset. An infantile, late-infantile and juvenile, as well as an adult form can be differentiated. The infantile form presents in infants younger than 6 months with hypotonia, seizures and blindness with characteristic cherry-red macular spots, usually leading to death before 4 years of age. The late-infantile and juvenile forms present in 2–10-year-olds, causing ataxia, spasticity and psychomotor deterioration—the cherry-red macular spots are, however, less common.

In adult patients the first manifestation is usually lower limb weakness (especially in proximal parts), caused by lower motor neuron dysfunction, with other symptoms including amyotrophy, fasciculations, cramps and abnormal electrophysiological results. The upper limbs are usually first affected later in the course of the disease. Other symptoms may include psychiatric abnormalities, cognitive impairment, dysphagia and dysarthria. A characteristic neuroimaging feature is cerebellar atrophy, which can be present even in patients without cerebellar symptoms. The disease has a chronic progressive course, leading to wheelchair dependence in half of all patients.

Patients with Sandhoff disease may also exhibit prominent dysautonomic features, including urine incontinence or constipation, especially those with adult-onset disease [52,53,54,55].

## Figures and Tables

**Table 1:** A summary of neuropathy types in different IEMs.

Neuropathy Type	Name of the Disease	OMIM	Affected Enzyme	Additional Comments
Acute neuropathies	Acute intermittent porphyria (AIP)	#176000	hydroxymethylbilane synthase (HMBS)	
	Tyrosinemia type I	#276700	Fumarylacetoacetate hydrolase (FAH)	
	PDH-deficiency	#312170	Pyruvate dehydrogenase complex	Mutation applies to the E1-alpha-polypeptide
Mononeuropathy, multifocal neuropathy	Tangier Disease	#205400	−	Transport protein-ATP -binding cassette transporter 1 (ABCA1) affected
Chronic axonal polyneuropathy	NARP/Leigh Syndrome	#551500/#256000	Mitochondrial H (+) -ATPase/complex I, II, III, IV or V	
	Leber Hereditary Optic Neuropathy	#535000	Respiratory complex I (NADH ubiquinone oxidoreductase)	
	alpha-methylacyl-CoA racemase deficiency	#614307	Alpha-methylacyl-CoA racemase	
	Phosphoglycerate dehydrogenase deficiency/ Phosphoserine phosphataseDeficiency/ Phosphoserine aminotransferase deficiency	#601815 #614023 #610992	Phosphoglycerate dehydrogenase, phosphoserine phosphatase, phosphoserine aminotransferase	Deficiency of these enzymes results in serine deficiency.
	Mitochondrial Trifunctional Protein (MTP) deficiency Long-chain 3-hydroxyacyl-CoA dehydrogenase (LCHAD) Deficiency	#609015 #609016	Mitochondrial trifunctional protein/long-chain 3-hydroxyacyl-CoA dehydrogenase	Both conditions may be caused by a mutation in HADHA gene.
	Brown-Vialetto-van Laere Syndrome Fazio-Londe disease	#211530 #211500 #614707	−	Defective protein-SLC52A3 or SLC52A2-riboflavin transporters
	Amyloidosis, Hereditary, Transthyretin-related	#105210	−	Mutation applies to the transthyretin
	Wilson’s Disease	#277900	Copper-transporting ATPase beta (gene ATP7B)	
	Cerebrotendinous Xanthomatosis	#213700	sterol 27-hydroxylase	
	Sensory ataxic neuropathy, dysarthria and ophthalmoparesis (SANDO)	#607459	DNA polymerase-gamma	
Chronic demyelinating polyneuropathy	β-mannosidosis	#248510	β-Mannosidase	
	Metachromatic leukodystrophy	#250100	Arylsulfatase A	
	Krabbe Disease	#245200	galactocerebrosidase (GALC)	
	Niemann-Pick B disease	#607616	acid sphingomyelinase (ASM)	
	Refsum Disease	#266500	phytanoyl-CoA hydroxylase (PHYH)	
Small fiber neuropathy	Fabry Disease	#301500	alpha-galactosidase A	
	Sandhoff Disease	#268800	hexosaminidase	Mutation applies to the beta subunit.
Lower motor neuron disease	Polyglucosan body disease	#263570	glycogen branching enzyme	
	Biotinidase deficiency	#253260	biotinidase	
